# Conceptual and normative approaches to AI governance for a global digital ecosystem supportive of the UN Sustainable Development Goals (SDGs)

**DOI:** 10.1007/s43681-021-00058-z

**Published:** 2021-05-06

**Authors:** Amandeep S. Gill, Stefan Germann

**Affiliations:** 1 Graduate Institute of International & Development Studies, Geneva, Switzerland; 2 Fondation Botnar, Basel, Switzerland

**Keywords:** AI, Governance, SDGs, Health, Digital, Commons

## Abstract

AI governance is like one of those mythical creatures that everyone speaks of but which no one has seen. Sometimes, it is reduced to a list of shared principles such as transparency, non-discrimination, and sustainability; at other times, it is conflated with specific mechanisms for certification of algorithmic solutions or ways to protect the privacy of personal data. We suggest a conceptual and normative approach to AI governance in the context of a global digital public goods ecosystem to enable progress on the UN Sustainable Development Goals (SDGs). Conceptually, we propose rooting this approach in the human capability concept—what people are able to do and to be, and in a layered governance framework connecting the local to the global. Normatively, we suggest the following six irreducibles: a. human rights first; b. multi-stakeholder smart regulation; c. privacy and protection of personal data; d. a holistic approach to data use captured by the 3Ms—misuse of data, missed use of data and missing data; e. global collaboration (‘digital cooperation’); f. basing governance more in practice, in particular, thinking separately and together about data and algorithms. Throughout the article, we use examples from the health domain particularly in the current context of the Covid-19 pandemic. We conclude by arguing that taking a distributed but coordinated global digital commons approach to the governance of AI is the best guarantee of citizen-centered and societally beneficial use of digital technologies for the SDGs.

The Covid-19 pandemic has been a rude awakening, and not just for public health systems. The virus exposed weaknesses in governance writ large. It also shone a stark light on the fragility of living standards; many developed countries discovered their poor for the first time and hundreds of millions of informal workers in developing countries lost access to work and plunged back into poverty. [1] The planet breathed a bit better for a while only to underscore the massive effort that is needed to prevent catastrophic climate change, mass extinction of species and more pandemics via wildlife squeezed out of its living space. The interconnectedness of all these challenges of health, employment, and environment is all too apparent. ‘Building back better’ comes near but does not quite capture the enormity of the task. [2]

Can digital technologies especially their latest presumably intelligent avatars be our savior? Could Artificial Intelligence (AI) fuelled by data help developing countries leapfrog to higher standards of living without straining the planet’s resources further and without compromising the agency and human rights of their citizens? Could such technologies help their richer cousins shift to more sustainable modes of economic growth while addressing inequity and exclusion? And could they help deliver high quality and affordable health care everywhere, and prevent future pandemics from destroying lives and livelihoods at the scale we are witnessing over the past year with the novel corona virus?

The answer depends on governance and a supportive global digital ecosystem [3].

## Conceptual foundations

### Human capacity and human agency

Before we address the key issue of governance, it is important to establish a clear conceptual foundation for digital technologies in the context of sustainable development. In the authors’ understanding, digital technologies are devices, platforms, data storage and processing architectures, algorithms, computing languages, communication protocols and standards that rely on the representation of information or data as discrete binary values. Information and communications technologies or ICTs, a co-terminus concept more used in the past, find mention in the United Nations Sustainable Developments Goals (SDGs) [4] or their predecessor, the Millennium Development Goals (MDGs) [5]. However, the context is narrowly defined in terms of access to ICTs [6]. This did not change much with the shift from the MDGs to the SDGs in 2015 except for an additional specific reference in SDG 9 to provide universal and affordable access to the Internet in least developed countries by 2020, and a passing acknowledgment that the spread of ICTs has great potential to accelerate human progress [7]. The reason was twofold: there was a lack of evidence of impact from the ground and there was a degree of conceptual confusion [8].

The focus has begun to shift since then from access and availability of technology and devices to inclusivity in use and benefit—this reflects the moral view of economist-philosophers, chiefly Amartya Sen, that freedom to achieve well-being is a matter of what people are able to do and to be, and thus the kind of life they are effectively able to lead [9]. The human capability approach embodies this thinking. It goes hand-in-hand with the notion of human agency, the ability of human beings to make choices and direct their own development. Humans have to be at the centre of design and deployment of digital technologies and their agency has to be respected in all interactions with machines especially as AI grows in importance.

Human capability is a solid conceptual foundation not only to understand and evaluate the development impact of digital technologies but also to bring development aspirations and human rights concerns together [10]. The Report of the UN Secretary-General’s High-level Panel on Digital Cooperation made an attempt at building synergy across these two domains using the lens of inclusiveness and digital public goods [11]. The G20 Ministerial Statement on Trade and Digital Economy of 8–9 June 2019 similarly recognized the cross-cutting nature of challenges posed by digitalization and affirmed the role of “Human-centered Artificial Intelligence” and ‘data for development’ [12]. Similarly, the 9 Principles for Digital Development have played a significant role in providing conceptual clarity for practitioners [13]. Further conceptual work in promoting a shared view of Development 2.0 enabled by digital technologies would be helpful in avoiding (false) dichotomies between control and promotion of use when it comes to governing the deployment of Artificial Intelligence for the SDGs or between providers of data and of AI solutions [14]. Without a shared conceptual vocabulary, the designers, the deciders and the practitioners of digitally enabled development would be like the proverbial blind men around an elephant. With one, they will have more confidence in their actions across subject domains and borders.

### Layered governance

The digital ecosystem is layered from its communication infrastructure and connectivity foundations to the logic layer, the applications layer and the content layer with myriad social and economic uses [15]. Governance too has to be layered like the content in form and function. Where international norms leave off, national regulation comes in, and where these regulations become blurred, ethical, and technical standards in the business-to-business and business-to-consumer space take over. This is subsidiarity in action where each layer of governance has a sovereign domain of impact but each one works in tandem with the other layers to achieve its purpose.

This is not the permissionless governance of distributed ledger technology enthusiasts nor is it novel decentralized collective governance for specific areas such as economics and finance [16]. Fundamentally speaking, this is analog governance adapted for the digital age. As has been shown in the context of regulating AI use in weapons systems, a tiered and distributed approach across three levels can act like sliding doors to keep policy responses up-to-date with technology development and prevent governance gaps from emerging [17]. Julian Eckl makes a similar argument in the domain of health by distinguishing governance interventions at the macro, meso and micro levels [18]. Interventions at the macro level such as minimum binding standards for the protection of personal data will be key to making the digital ecosystem safe and inclusive, for instance, for the collaborative and publicly beneficial use of health data. Interventions at the meso level can be tailored to specific contexts such as the sharing of disease-specific data during a pandemic. Finally, interventions at the micro level can help individuals and collective agents to understand the (health) implications of their data-related decisions.

Layered governance goes hand-in-hand with the avoidance of centralization of data. Centralizing datasets and interoperable data architectures—with the exception of a few critical national databases—enhances the risk of hacking and misuse. Instead, they can sit in a distributed hubs and spokes geometry and contribute to the solving of global challenges such as the growing resistance to anti-microbials due to over-prescription of antibiotics, without centralizing data while building up local capacity on using data science to address context-specific challenges. This reinforces inclusiveness and agency as opposed to a neo-colonial dichotomy of ‘problem-owners’ in data-rich but algorithm and compute capacity poor geographies versus ‘problem-solvers’ in technologically advanced countries. Going hyperlocal with data means that there are no ‘big data’ lakes or pools that can be exploited by powerful actors. Instead, the focus is on data flow and data use in a distributed manner with the long-term goal of data and AI, say for health, for the public good.

## Normative approaches

### Human rights first

If conceptually we are still in transition from ICTs and mobile devices in development to a more holistic framework of “digital development” [19] and distributed governance, normative approaches also show a significant lag with regard to what is required. There was misuse in the ICTs era with malware and criminality. However, the consumer protection and regulatory challenges of the 1990s pale in comparison to what we have witnessed over the past decade. For instance, Philip Alston, Special Rapporteur on extreme poverty and human rights, argued forcefully in 2019 that “digital data and technologies … are used to automate, predict, identify, surveil, detect, target and punish” welfare recipients [20]. In other cases, national security agencies field digital technologies to monitor civil society activists, opposition groups or terror or crime suspects.

At the same time, private social media platforms and digital commerce enterprises have proliferated, privately held datasets have grown and algorithms to extract intelligence from personal and public data have improved in sophistication. The power and profit that accrues to those who dominate key sections of the digital value chain today is enormously superior to the internet businesses of the 1990s. Network effects drive consumers and revenues to a small number of digital giants creating vast asymmetries in market power.

What does this mean for norms on human rights such as those enshrined in the Convention on the Rights of the Child? [21]. These have traditionally been addressed to States, which have a duty under international law to protect them. The circle of impact of private companies, say in mining or forestry, was limited. This has changed with digitalisation. Online platforms developed privately function now virtually as global digital public infrastructure, and algorithms designed in one place can impact decisions and behavior in other geographies. And unlike other industries, digital companies can impact the rights of millions at once. That they operate seamlessly across borders complicates government efforts to work with them to protect the human rights of users within their jurisdiction.

Digital platforms and tools have been used to promote terrorism and violence against the vulnerable. They have been used for mass surveillance and to harass and intimidate journalists and civil society activists. Wittingly or unwittingly, they have been party to the purloining and exploitation of personal data, and they have provided anonymity to a range of bad actors from scamsters to pedophiles. Children are particularly vulnerable with a massive uptake of digital devices and apps by them in the last 10 years. Digital technologies directed at the datafication and dataveillance of young children are growing in sophistication, with potentially life-long negative ramifications [22].

Normatively speaking, therefore, a first-order task is the strengthened implementation of existing international standards on human rights. There can be no digital carveout or exceptionalism on universal instruments on human rights; they apply online as well as offline. National laws and regulations must be reread in the light of advances in digital technology particularly AI replying on personal data to enhance protection; equally international organizations and forums must make a fresh interpretation of existing applicable human rights law to ensure that digital technologies are not used to erode human rights or avoid accountability. The preparation of a draft General Comment on children’s rights in relation to the digital environment by the Child Rights Committee is a case in point [23].

Further, robust and systematic human rights impact assessments of all digital interventions throughout their full life cycle should be conducted to ensure that adherence to human and child rights is more than lip-service [24]. The next sub-section elaborates this aspect further.

### Multi-stakeholder smart regulation

Further, upholding norms in the digital age requires better coordination and communication between governments, technology companies, civil society and other stakeholders. This is sometimes called smart regulation or ‘new governance’ [25]. An illustrative problem is how to encourage the responsibility of the private sector for the human rights impact of their products and services. Companies have often reacted slowly and inadequately to learning that their technologies are being deployed in ways that undermine human rights. Therefore, we need more forward-looking efforts to identify and mitigate risks in advance: companies could consult with governments, civil society and academia, for example, to assess the potential human rights impact of the digital technologies they are developing. Risk assessment must be followed by ongoing due diligence and responsiveness to events, for which better bridging is needed to human rights expertise available in civil society and international forums; and the companies themselves need to employ and train their own human resource.

Such smart regulation should extend also to upholding norms on preventing monopolies, protecting consumers and maintaining a vibrant innovation ecosystem for digital startups and small companies. In India, civil society and tech pioneers played a role in bringing 90 odd manufactures of drones and the civil aviation authorities together to craft a new paper-less regulatory scheme for commercial drones before the largescale deployment of such technology [26]. *Samaj, Sarkar* and *Bazaar* or civil society, government and business, respectively, came together to make this happen.

Such multi-stakeholder coordination can be routinised through well-designed regulatory sandboxes, which are a growing feature in discussions on AI governance. These can allow developers to test algorithms and transition from in silico to real life situations. There can be other dynamic regulatory mechanisms based on data models such as the use of digital “avatars” for virtual pre-tests before real life testing. Caution must, however, be exercised in extending insights from small groups of users to large scale deployment since governance requirements may shift as complex societal interactions emerge. In particular predictive systems in certain settings such as law enforcement, justice, education and employment may show their true colors much later. Therefore, stakeholder engagement should be maintained beyond the sandbox setting for post-use analysis at regular intervals.

### Privacy and protection of personal data

Protection of privacy is a particularly important area for smart regulation or agile governance. The public health response to Covid-19 highlighted a serious lack of consensus on privacy protecting proximity tracing using digital apps. There was a trust-deficit between users on the one hand and governments and/or private companies on the other leading to low adoption rates [27]. Collaborative and transparent design of standards with academia playing a bridging role between governments and companies could have been a key enabler. A similar approach did succeed with regard to the rapid development and emergency approval of Covid-19 vaccines [28].

Privacy-preserving or enhancing technologies are only one part of the solution. Personal data portability to promote a healthy competition on preventing privacy violations is another. Empowerment of users through data literacy, greater transparency of data use and clearly explained consent procedures is the third and perhaps the most important part of the answer. Governments will be tempted to pick national winners on data privacy but should avoid the temptation so as not to create future monopolies and avoid friction across borders on an issue that should enjoy universal consensus. Instead, they can focus on building enforcement capacity and data literacy locally—which current normative frameworks tend to leave in individual hands—while collaborating with their counterparts on cross-border violations of personal data protection. Cyber security capacity to protect data from malicious attacks should be a critical subset of these national efforts.

There are painful trade-offs between the welfare impact of sharing and the benefits of privacy [29]. As the UNSG’s High-level Panel on Digital Cooperation noted companies, governments and civil society need to agree to clear and transparent standards that will enable greater interoperability of data in ways that protect privacy while enabling data to flow for commercial, research and government purposes, and supporting innovation to achieve the SDGs [30]. The recent experience with Covid-19 contact tracing apps underlines that such standards should prevent data collection going beyond intended use, limit re-identification of individuals via datasets, and give individuals meaningful control over how their personal data are shared.

### A holistic approach to data

We make a mistake by framing data governance almost exclusively in the context of misuse of data. This ignores obstacles to the secondary use and sharing of data for societal benefit. Such an approach also risks alienating dynamic geographies of digital innovation in Asia and Africa who fear missing out on another leapfrogging opportunity and whose cooperation would be essential for the success of a global governance approach. If data governance looks like a list of do’s and don’ts to policymakers and entrepreneurs in the Global South, they would wonder at the motivation behind such a narrow focus on *misuse* and not on *missed* use or on *missing* data for policymaking and technology solutions. To be successful and sustainable, AI governance, seen in conjunction with data governance as we argue later in this piece, has to integrate these three ‘Ms’ thoughtfully. The right balance between closed data (for personal privacy or intellectual property reasons) and open data (for social and economic benefit) might vary from country to country. Some geographies might require incentives to encourage investments in data collection, curation, use and reuse while others might require incentives to encourage opening of existing datasets and data sharing across public and private sectors. Nonetheless, all three perspectives have to be balanced carefully in regulating data collection and use.

The health domain provides a good example in the context of a holistic approach to data. Millions of births and birthweights are still not registered or are registered improperly across the globe. Death certificates are either not filled out or contain too generic a description, say respiratory failure, of the cause of death thus denying an opportunity for advancing understanding of the underlying causation. Then there is missed use; the data on prescription of antibiotics is there but it is lying in the data warehouse of an insurer or a hospital and the opportunity to study, antimicrobial resistance, for example, is lost. Another common problem is that data for health is sitting in non-interoperable silos and does not come together at the right time for the right people.

The focus on missing data and missed use of data need not detract from preventing the misuse of data, vital to promoting long-term trust in digital health solutions. On the contrary, building up new datasets in responsible ways and devising data architectures to be interoperable across different communities of practice is an opportunity to reinforce good governance on avoiding misuse of personal data and the institutionalization of bias in AI algorithms.

### Global collaboration

Digital technology that can be deployed seamlessly across the globe and algorithms that can pull in data from anywhere cannot be governed by a single government or organization alone. Products and services based on these technologies can quickly reach global scale of adoption without validation in specific and diverse contexts. Therefore, a global response is must. The essence of ‘digital cooperation’, which is finding its place alongside multi-stakeholder internet governance as the term of choice in the algorithmic age, is about government, private sector and others coming together to promote guard rails and common rails. The UN Secretary-General’s High-level Panel on Digital Cooperation has proposed three models of collaborative digital governance; the subsequent Road Map for Digital Cooperation notes the continuation of consultations on the various models and proposes some measures for improving the functioning of the existing multi-stakeholder Internet Governance Forum [31].

In digital cooperation, collaborating partners use existing values and principles or discover them in their social, political, and economic context through a process of dialog. This helps them align incentives and find common purpose. They then commit to cooperate through a governance modality and develop mechanisms as well as capacity to promote responsible use and global governance of digital technologies.

Globally, a holistic approach to data noted previously translates into a modular governance approach built on a strong common foundation of shared principles and values. Significant work has already been done on the latter [32]. In addition to shared principles, common industry standards would help build interoperability with national, international, and industry standards acting in concert to provide flexible responses to governance challenges [33]. A critical missing piece is governance of international data flows, which for the foreseeable time might have to rely on ad hoc arrangements. In time, consensus could emerge on data interoperability in areas such as global health, or subset domains within health such as epidemiology, which then would help ease agreement in more contentious domains such as trade. The cross-border use of contact tracing apps and the mutual recognition of digital vaccination certificates for travelers are two emerging areas of global collaboration [34].

Another possible area of consensus could be a global framework on health data governance to help harmonize national efforts [35]. Research institutions and neutral platforms free of geopolitical or commercial agendas can play an important role in building consensus. It is encouraging to see several new initiatives in this regard [36].

### Basing governance more in practice, in particular, thinking separately and together about data and algorithms

There is a plethora of industry, civil society, even some government initiatives on AI governance. Principles and codes are mushrooming but there is lack of cohesion in their adoption and implementation; absence of ethical analysis and implementation strategies are two of the significant gaps [37].

A reason for the surfeit of abstraction and lack of viable implementation strategies is that the layer of practice of AI in concrete domains such as health, education or the environment on which to base governance responses is still too thin for sustainable and (globally) scalable models to emerge. Instead of top-down broad approaches to the governance of AI and data, the practice-based governance of these technologies in specific domains such as health could give us a foundation of evidence on which to build thoughtful governance models [38]. It would encourage ground-up innovation in governance. A tighter loop between practice and norms, as illustrated with a schema for health AI governance later in the next section, would also help reduce the lag between technology development and normative guidance.

An important issue for practice is the distinction as well as the close relationship between algorithms on the one hand and the data used to build them on the other. Data are structured and unstructured information and observations stored and manipulated digitally for calculations, reasoning and qualitative insights. Datasets are structured data specific to a domain or a use context. While traditional software writing involved manipulating data to reach desired outcomes, AI development uses historical outcomes dynamically to build software models for future outcomes. The assessment of outcomes—past or future—is subjective; human behavior is therefore the third critical dimension of autonomous intelligent systems.

Two recent experiences with the use of algorithms, one in UK in the area of education [39] and another in the United States in the area of health [40], have underlined the complex interplay between data, algorithms and human behavior. As designs and prototypes move to practice, who takes responsibility for bad decisions, and who bears the liability for costs to third parties? To what extent is the outcome attributable to the algorithm, and to what extent is it the result of incomplete, poorly selected or biased datasets used to build the AI model?

The wag may have it right when artificial intelligence is juxtaposed with “natural stupidity”. The governance problem goes above and beyond unbiased data, transparency and explainability of algorithms. Public officials need to understand the limitations of autonomous intelligent systems, and the different roles played by statistical methods, datasets and algorithms in such systems. They need to prepare for end-to-end governance of autonomous intelligent systems rather than simply AI or data governance. Identifying the limitations and trade-offs at each stage of system development, reinforcing human responsibility, and accountability for use, and providing for post-deployment assessments should be integral to this lifecycle approach to governance.

The UK Competition & Markets Authority (CMA) has highlighted the challenge for regulators in a recent paper on what it calls ‘algorithmic systems’—a larger intersection of the algorithm, data, models, processes, objectives, and how people interact and use these systems. Regulators need to be trained and need to develop a set of techniques to audit the systems on an ongoing basis, assess harms and remedy them without necessarily having access to the code and the training datasets. They can also help develop standards and facilitate accountability by supporting the development of ethical approaches, guidelines, tools and principles [41].

## A global digital commons approach to AI governance

How do we bring the conceptual foundations and the normative approaches together? How do we ensure that there is no ‘jurisdiction shopping' and industry standards, national regulation and international norms come together in a mutually supportive digital ecosystem? How do we make sure that governance solutions do not have a centralizing, self-selecting feel, and entry barriers for participation in governance discussions do not disadvantage the already marginalized?

We are not starting from scratch. Over the years, governments, technology associations, businesses, and civil society have come up with various mechanisms to govern digital technologies. The Geneva Internet Platform (GIP) lists more than 1000 such mechanisms under various categories from international conventions and court judgements to standards and recommendations [42]. There is considerable support for a toolbox approach to AI governance spanning universal principles set by international forums, national and regional regulation, non-binding industry norms, and best practices as well as individual users’ settings and preferences [43].

There is also emerging interest in a global digital commons approach to spread the benefits of digital technologies more widely and prevent another ‘tragedy of the commons’ through misuse. As argued in the report of the UN Secretary-General’s High-level Panel on Digital Cooperation, apart from promoting the SDGs and addressing social harm, a digital commons architecture can create dialog on emerging issues and communicate use cases and problems to be solved to multiple stakeholders [44]. The muti-stakeholder tracks or platforms constituting this architecture could also disseminate new data and evidence about the impact of artificial intelligence and other emerging technologies, thus making discussions on governance more ‘factful’. As part of this feedback loop, they can also help stakeholders such as AI developers on the ground assimilate soft governance norms at an early stage in their design and development work. Finally, they could make the case for new investments into collaborative research and development of data architectures and AI as well as related infrastructures and capacity building.

**Fig. 1:**
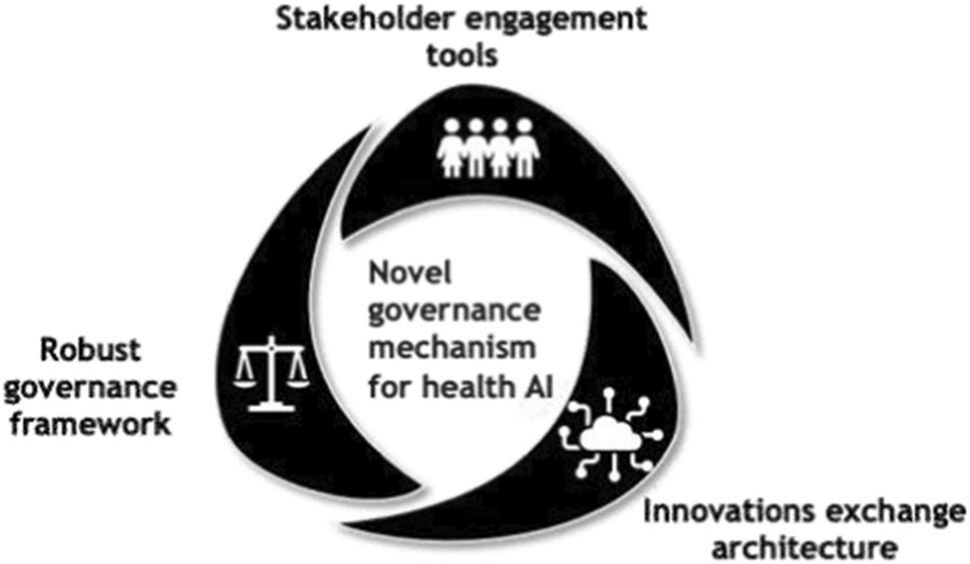
A novel governance mechanism for health AI: Source: I-DAIR, 2021

A schematic for a governance mechanism for a health AI-related track inside such a digital commons architecture is described below. This three-part governance mechanism can be applied globally to the data and AI tools proposed to be used for health research, healthcare, and health promotion through a network of collaborating institutions based in different national jurisdictions (Fig. 1).

Its three principal components and their main functions are:

1. Robust governance framework with:
  A data protection and data security core;
  A data process to ensure responsible and fair use of data;
  A risk-based classification of sensitivity to avoid misuse of data;
  Context-specific (re)discovery of international principles, mechanisms, and norms.
2. Stakeholder engagement tools that enable:
  Mapping to identify and engage stakeholders innovatively;
  Capturing stakeholder contributions to AI solutions and their governance throughout the development process;
  Monitoring which allows stakeholders to highlight issues post-deployment;
  Appropriate communication strategies to demonstrate bottom-up problem solving and trustworthiness of AI across all stakeholders.
3. Solutions exchange architecture that:
  Enables exchange of governance assets in the form of best practices in co-creation, stakeholder consultations, inclusiveness, data protection principles, and regulatory mechanisms.
  Enables pooling of data power through common problem definition and research design without aggregating data;
  Promotes governance learning from diverse practice of AI for health, and a shared stake in global governance principles, mechanisms, and norms and best practices in stakeholder co-creation, consultation, communication.

For the mechanism to be applicable across different tiers of use and geographies, it cannot be in the form of a single regulatory standard. Instead, it is devised as a community-facing governance innovations exchange anchored in a robust set of principles, mechanisms, and norms.

## Conclusion

In 2030, of the global population of 8.55 billion, 39% or 3.31 billion would be young people under 25 years of age and 24% or 2.03 billion would be children under 15 years of age. Almost one-third of them would live in Africa. These young people, many of whom may well be digital natives, could be a tremendous asset given the right educational and economic opportunity. However, if existing inequities continue to fester, exacerbated by a lack of access and agency driven by the persistent 'digital divide', the incredible opportunity of a skilled and engaged 'youth bulge' would be missed, resulting in a lost generation, consuming digital products made by a select few, and not participating meaningfully in the digital transformations under way [45].

A human rights based normative framework offers not only protective, defensive rights but also entails solidarity entitlements such as participation in scientific progress and technological advancements and rights such as the right to development and the right to health. Urgent efforts are required both to enhance protection of human rights as also to promote access to opportunity, quality content and the skills needed to thrive in a world of AI and data systems.

Conceptually, the human capability pillar allows us to link the human rights centered normative approaches to the practice of digital development. Additionally, the layered governance concept links global norms to local practice. Norms percolate down and governance innovation moves up from the edge to the center. Data use and algorithmic development are not centralized and the risk of neo-colonial dichotomies between ‘problem-owners’ and ‘solution-providers’ is reduced.

The global digital ecosystem is distributed across layers. No one can rule it all. Governance too has to be layered using appropriate tools from the agile governance toolbox at different layers. These layers have to be connected by communities and networks of norm developers and practitioners with light touch coordination by multilateral and multi-stakeholder platforms. This shift from ‘command and control’ to ‘connect and cooperate’ is at the heart of the conceptual and normative framework for a globally coordinated digital commons approach to AI governance presented in this article.
